# Index Modulation Embedded in Type I Waveguide Written by Femtosecond Laser in Fused Silica

**DOI:** 10.3390/mi12121579

**Published:** 2021-12-18

**Authors:** Jing Lv, Razvan Stoian, Guanghua Cheng, Kedian Wang

**Affiliations:** 1 School of Artificial Intelligence, Optics and Electronics (iOPEN), Northwestern Polytechnical University, Xi’an 710072, China; lvj1228@126.com; 2 Laboratoire Hubert Curien, UMR 5516 CNRS, Université de Lyon, Université Jean Monnet, 42000 Saint Etienne, France; razvan.stoian@univ-st-etienne.fr; 3 State Key Laboratory for Manufacturing System Engineer, Xi’an Jiaotong University, Xi’an 710054, China

**Keywords:** femtosecond laser, fused silica, waveguide, refractive index change, periodic pattern, mechanical perturbation

## Abstract

Slit-shaped laser beams focused in bulk optical materials can realize embedded waveguides with circular cross sections consisting of positive index change type I traces. In these kinds of waveguide traces, a peculiar periodical refractive index modulation was observed in type I waveguides with two different femtosecond lasers. The direction of refractive index modulation can be controlled with the slit configuration, and its period can be controlled by mechanical perturbation of the stages and the scanning speed. We argue that platform perturbation and dynamical thermal transport processes during the scan are generating factors in the appearance of this modulation. The embedded microstructures in waveguides can provide spectrum modulation, which may have potential applications in optical sensing, filtering, and phase control.

## 1. Introduction

Femtosecond lasers are considered powerful tools for inducing nonlinear absorption and microscopic structural changes with precise energy deposition inside transparent materials, primarily due to their ultrashort pulse duration as well as ultrahigh peak power [1,2,3,4]. In investigating the interaction between femtosecond laser pulses and matter, fused silica has been, several times, a common platform for studying ultrafast phenomena and for proposing applications and systems, such as optical diffraction devices, microflow devices, and optical waveguide devices. This is motivated primarily by its excellent optical, mechanical, and chemical characteristics [5,6,7]. Recently, the appearance of periodically nano- and microstructures induced by femtosecond laser pulses inside silica glass has become a very important topic since it promises the fabrication of polarized sensitive devices [8]. Therefore, it is important to understand the formation of laser-induced periodic surface structures and nanogratings. The induced gratings consist of narrow layers of lower refractive index, with an interlayer period of approximately λ/2n (where λ is the free-space wavelength, and n is the refractive index of the fabrication material) and dependent on laser exposure [9]. Furthermore, controlled dependencies on laser parameters allowed fine-tuning birefringence and retardation by adjusting specific fabrication parameters [10], which provides polarization control in monolithic 3D-integrated systems. In particular, the period of self-organized grating structures was controlled by the pulse energy and the number of irradiated pulses [11]. The typical nanogratings reveal strong bond breakage and deficit of oxygen [12,13], with void-like morphologies (type II), and seem to be the result of regular field enhancement due to scattering and interference [14]. On the other hand, the self-organized microstructures with a period larger than laser wavelength have been demonstrated using a high-repetition-rate femtosecond laser [15,16], whose possible explanation concerns the hydrodynamics instability. The pearl-chain structures with periodically distributed bubbles, having optical waveguiding properties, were fabricated at a 10 MHz oscillator due to rapid cooling and solidification of the molten material [15]. Similarly, the dynamical formation of self-organized bubble patterns was explored in the cumulative regime, including the condition for identifying transitions from chaotic to periodic regimes [16].

In this paper, we focus on a new type of modulated microstructures embedded in type I waveguides (type I waveguide is obtained for relatively low laser-power density and short pulses, where an increased refractive index (10^−4^–10^−3^) is observed in the irradiated regions) written by a 50 kHz repetition-rate femtosecond laser inside fused silica. Periodic patterns of an index modulation period can be produced, organized, and controlled in waveguide structures by changing laser processing parameters. The mechanical perturbation of the motion platform and instabilities in uniformly evacuating the energy from the interaction regions are revealed as trigger factors for modulated microstructures.

## 2. Experimental

### 2.1. Materials

A polished parallelepiped fused silica block (Corning 7980) with a size of 20 mm × 10 mm × 4 mm was used in our experiments. Before laser treatment, the glass was cleaned ultrasonically with industrial ethanol.

### 2.2. Fabrication of Optical Waveguides

We reproduced the experiment with two femtosecond laser systems separately: one is a Ti:Sapphire regenerative amplifier femtosecond laser system (Phidia, Uptek Solutions, Bohemia, NY, USA) delivering 50 kHz repetition rate Gaussian laser pulses of pulse width τp = 140 fs at 780 nm central wavelength; the other one is a Yb:KGW femtosecond laser system Pharos^®^ (Light Conversion, Vilnius, Lithuania) which has a 200 kHz maximum repetition rate, 225 fs pulse duration, and is operated at 1030 nm central wavelength. The laser energy was attenuated to the desired value by a half-wave plate and a Glan polarizer. The combination of a couple of cylindrical lenses and a slit can exploit the advantages of both beam-shaping components for flexibly controlling the intensity distribution on the premise of low-loss incident laser, h, has been used for beam shaping to produce waveguides with circular cross sections in transverse section [17,18]. Then, the truncated beam was focused via a 20× (0.42 NA in Mitutoyo and 0.4 NA in Motic for 780 nm and 1030 nm laser wavelength, respectively) microscope objective, leading to a reshaped disk-like confocal region. Fused silica materials (Corning 7980) were used as the specimens and were moved transverse to the incident laser beam by a computer-controlled 3D translation stage (Physik Instrument stage served for Phidia laser, and Aerotech stage served for both Phidia and Pharos lasers). Both single-scan and multiscan writing techniques were applied to obtain the waveguides with low propagation loss. It has to be noted that all waveguides had linear shapes when the glass sample was moving along the single axis (corresponding to Physik Instrumente M-111.1DG and Aerotech ANT 130 stages, respectively). Several important processing parameters were investigated in our experiment, including pulses energy ranging from 0.8 to 3.0 μJ, writing speed from 25 to 600 μm/s, focus depth from 50 to 300 μm, and the changed polarization direction of the injected laser.

### 2.3. Characterization and Measurement

After the waveguides were written, the near-field modes of the guided light were captured using a 5× microscope objective onto a CCD camera. The waveguide tracks were imaged by a positive optical phase-contrast microscope (PCM, BX51, Tokyo, Japan), which provides a qualitative assessment of the refractive index changes with positive and negative changes visible, respectively, as black and white zones, respectively, on a gray background. Micro-Raman spectra were recorded using a micro-Raman InVia Reflex (Renishaw, London, UK) spectrometer, with a laser beam at an operating wavelength of 514 nm. To obtain distinct etching patterns, the microstructures were exposed with polishing treatment and then etched in a weak aqueous solution of hydrofluoric acid (HF, 2% by volume) for 15 min. By using the scanning electron microscope (SEM, S3000N, Hitachi, Tokyo, Japan) imaging, the regular oscillating patterns modified by femtosecond laser writing were investigated.

## 3. Results and Discussion

### 3.1. Effect of Processing Parameters on Waveguide Track with Periodic Patterns

**Figure 1:**
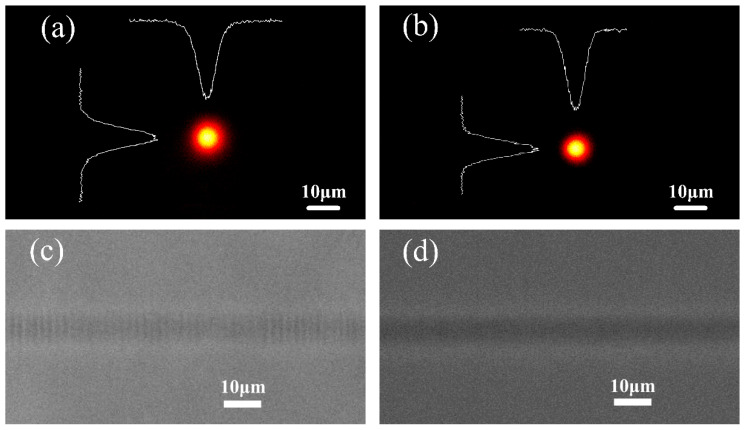
Near-field mode images of the optimal single-scan (**a**) and multiscan (**b**) waveguides in fused silica; PCM images of the corresponding single-scan (**c**) and multiscan (**d**) waveguide tracks.

Propagation loss measurements were carried out on all the waveguides fabricated by the Ti:Sapphire femtosecond laser and PI stage through coupling fibers to the waveguides. The output power after propagation through the waveguide with a different length was measured based on the cut-off method. It was found that the optimal single-scan waveguide with the lowest propagation loss of about 0.6 dB/cm at 976 nm was fabricated using a translation velocity of 100 μm/s and pulse energy of 1.6 μJ. For multiscan fabricated waveguides, the optimal processing parameters were a scanning speed of 500 μm/s, pulse energy of 0.9 μJ, and 8 repetitive scans, yielding to a propagation loss of 0.3 dB/cm. Figure 1 shows standard images of femtosecond laser’s written optimal waveguides in fused silica. In Figure 1a,b, the guided near-field modes at 976 nm are captured for the optimal single-scan and multiscan waveguides, respectively. The single-scan waveguide had a horizontal diameter (full width at half maximum) of 7.5 μm and a vertical diameter of 7.7 μm. The mode field of the multiscan waveguide was symmetric, with a horizontal diameter of 6.4 μm and a vertical diameter of 6.1 μm. Therefore, the beam-shaping method used here is rather successful in delivering quasi-circular profiles. The reason for the difference between two mode fields lies in the obtained maximum refractive index change that was measured to be 1.4 × 10^−3^ and 2.1 × 10^−3^ at 976 nm for single-scan and multiscan waveguides, respectively (the refractive index distribution is obtained through the reverse deduction of the scalar wave equation from near-field mode distribution). The PCM images in Figure 1c,d reinforce this evidence in which the single-scan waveguide track has a lighter color and smaller index contrast, leading to a larger guided mode. What needs to be highlighted is that positive index changes appear dark in the PCM images, while light zones indicate a negative index. Interestingly, there are regular oscillatory patterns inside the single-scan waveguide track, compared with the multiscan waveguide, as shown in Figure 1c. Therefore, oscillatory patterns are linked to nearly periodic type I refractive index changes induced in the space by femtosecond laser modification, with a period in the range of 2 μm. The onset of this periodic refractive index change may be one of the reasons for the higher propagation loss of the single-scan waveguide, resulting in the periodic perturbation and the rugged interfaces. Consequently, some incident light might be dissipated through scattering with nonuniformities. The better guiding in the multiscan waveguide is mainly related to better mode confinement, and the roughness of the guide interface is smoother. In addition, because the normalized frequencies of these two waveguides are different, the less-confined mode is another reason for propagation loss.

**Figure 2:**
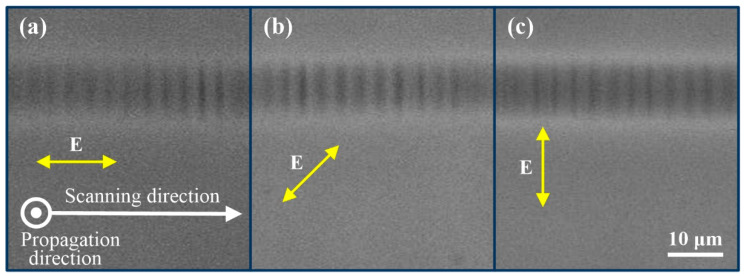
PCM images of waveguide tracks using different polarizations for the laser pulses at the scanning speed of 350 μm/s and pulse energy of 2.4 μJ: polarization angle of (**a**) 0°, (**b**) 45°, and (**c**) 90° with the horizontal direction.

**Figure 3:**
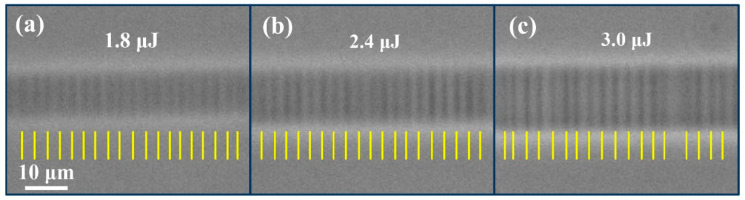
PCM images of waveguide tracks using horizontally polarized laser pulses with different pulse energies at the constant scanning speed of 250 μm/s: (**a**) 1.8 μJ, (**b**) 2.4 μJ, and (**c**) 3.0 μJ.

The influence of laser processing parameters on the characteristics of oscillatory patterns was further investigated using the single-scan technique, including laser pulse energy, polarization direction, and scanning speed. The laser polarization angle was rotated to be 0°, 45°, and 90° with respect to the scanning direction by rotating the other half-wave plate in the experiments. Our result, shown in Figure 2, demonstrates that laser polarization has no impact on the orientation of the periodic patterns. However, a previous work exhibited that both laser polarization and scanning direction have influenced the self-organized planes arising from the quill or nonreciprocal writing effect [19]. These nanogratings were linked to a process of light scattering and interference [20]. The independence to polarization here, in addition to the spacing, indicates that a different formation mechanism is at work. We observed a relatively smooth waveguide trace when the pulse energy was no more than 1.0 μJ, at the fixed scanning speed of 100 μm/s. With an increase in the pulse energy, the periodic patterns become apparent. The phenomenon is in agreement with the observation of Figure 1d: no observably periodic patterns are seen at a low energy value of 0.9 μJ using the multiscan technique, because of homogenizing treatment for multiple scanning and lower index change induced by lower pulse energy. The average period is little influenced as the pulse energy is higher than 1.4 μJ at the scanning speed of 250 μm/s, as shown in Figure 3. Moreover, the robustness of periodic structures becomes worse with the random overlay of multiperiod patterns and short-range disorder when waveguides were formed by larger pulse energy of 3.0 μJ, as shown in Figure 3c. The glass matrix was damaged with type II periodic patterns inside when the laser energy further increases. The orientation of the damaged periodic pattern is perpendicular to the pattern in type I waveguide, and the period is in a magnitude of micrometer, which is not the focus in this work and is not discussed here.

**Figure 4:**
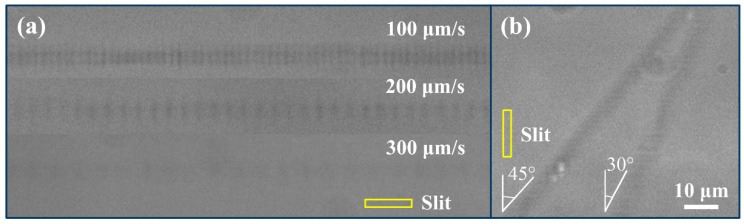
PCM images of waveguide tracks fabricated by 1030 nm femtosecond laser system with (**a**) different scanning speeds and (**b**) different scanning directions.

**Figure 5:**
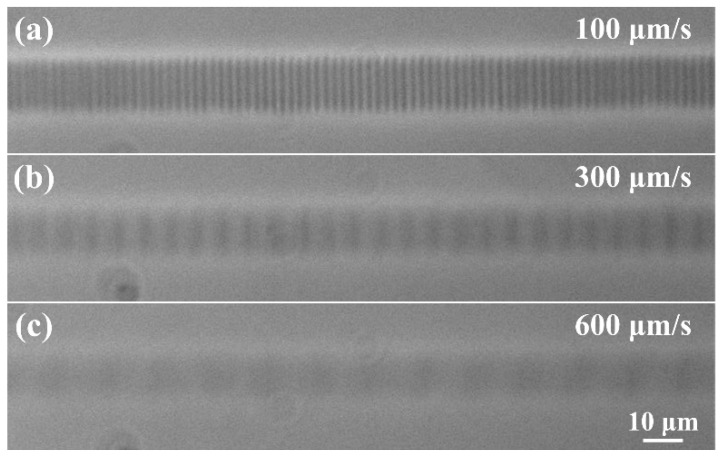
PCM images of waveguide tracks fabricated by 780 nm femtosecond laser and Aerotech stage system with different scanning speeds: (**a**) 100 μm/s, (**b**) 300 μm/s, and (**c**) 600 μm/s.

Then, we repeated the experiments to investigate the influence of pulse energy and polarization direction on the characteristics of oscillatory patterns with Yb:KGW laser processing system. It was also proved that laser polarization and pulse energy have little impact on the orientation and the period of oscillating patterns, respectively. Furthermore, Figure 4a,b show the longitudinal profiles of the single-scan waveguides with different scanning speeds and scanning directions. In Figure 4a, the overall amplitude of the refractive index change and the cross-sectional size of waveguides decreased as the scanning speed increased. The periodicities of these modulated patterns were 2.2, 4.3, and 6.5 μm, at the scanning velocities of 100, 200, and 300 μm/s, respectively. This indicates a linear scaling between the dose and the period. Interestingly enough, the time interval between processing each neighboring pattern is ~22 ms. Observable pattern-embedded or smooth, structureless waveguides were not obtained with the scanning speed higher than 600 μm/s due to the decrease in laser energy per unit volume. This experiment was repeated with the Ti:Sapphire femtosecond laser and Aerotech stage, shown in Figure 5. The periodicities of these modulated patterns fabricated were 2.2, 6.6, and 13.1 μm, at the scanning velocities of 100, 300, and 600 μm/s, respectively. The same time period was obtained, which shows that the fabrication of modulated patterns can be well repeated. At the same time, it is demonstrated that laser wavelength and pulse width have no impact on the periodicity of the modulated patterns. The relative orientation between the confocal geometry and the slit is also of importance. In Figure 4b, the angle between the scanning direction and the vertical direction is 45 degrees and 30 degrees, respectively, while the position of the slit is fixed and shown in the yellow box, indicating that the beam was shaped in the horizontal direction. This shows that the direction of modulated patterns has no link with the scanning direction. Then, by comparing Figure 4a,b based on the above conclusion, we can see that the direction of periodic modulations is always parallel to the slit-beam-shaping direction. We recall that the focused spot through slit shaping is similar to a disk that has expanded in the horizontal direction and remains unchanged vertically, shown in Figure 4b. As a result, the waveguide tracks were produced similarly to imprinting the footprint of the disk in a piece-by-piece manner. However, the angle between the scanning direction and the long axis of the slit cannot be too large; otherwise, the influence of rectangular aperture on the aspect ratio of femtosecond written waveguides will lose its meaning. For instance, the waveguide track had little type II damage when the included angle between the writing direction and the long side of the slit was 45 degrees. This result reveals that the oscillatory index patterns fabricated by 1030 nm femtosecond laser are less visible than those generated by 780 nm femtosecond laser. The energy window for type I waveguide and the increase in refractive index change become smaller: No refractive index changes are generated with lower energy pulses, and the irradiated region is catastrophically damaged with higher energy due to the longer pulse duration of the Yb laser. The pulse width of Yb:KGW femtosecond laser is 225 fs, close to the upper limit of the pulse width for fabrication of type I waveguide in fused silica. In addition, from Figure 5, we can see that the larger modulation period is obtained with a larger scanning speed, to extend the modulation range under the premise of a great increase in the overall index change in waveguide track.

### 3.2. Raman and SEM Analysis

**Figure 6:**
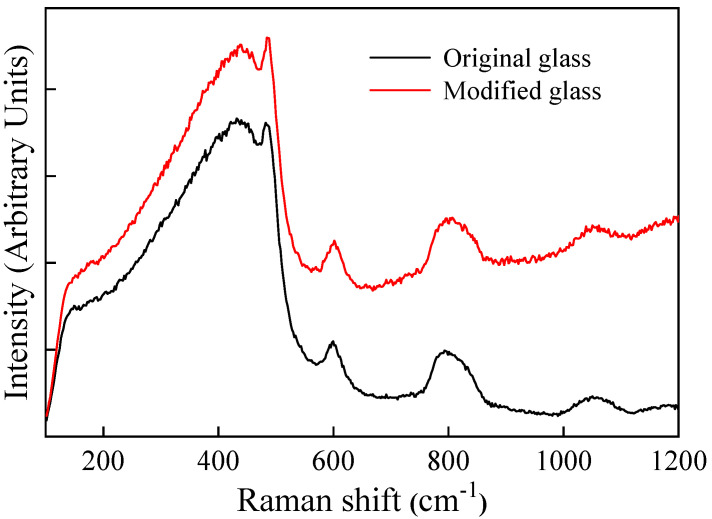
Raman spectra for waveguide in fused silica fabricated by femtosecond laser.

Figure 6 compares the Raman spectra of the original glass and the modified glass under the irradiation condition of the pulse energy of 1.6 μJ and scanning speed of 100 μm/s. One can see that the fluorescence effect of laser irradiation is obvious, due to the formation of nonbridging oxygen hole centers after the breakage of the Si-O bond [21]. There is no significant variation in the Raman peak ratio across the modified region, which is in agreement with the result in Ref. [22]. The two main causes were that (1) the lower energy deposition in the focal volume induced a small incremental change of three-membered and four-membered ring structures, and (2) the polishing treatment before Raman analysis increases the surface pressure, resulting in the densification of the whole cross section of the waveguide. In addition, the Raman linear mapping on the oscillatory-patterns-embedded waveguides was generated. Unfortunately, the refractive index change of the periodic oscillations is too small to reflect the change in the intensity and shift of the characteristic peaks.

**Figure 7:**
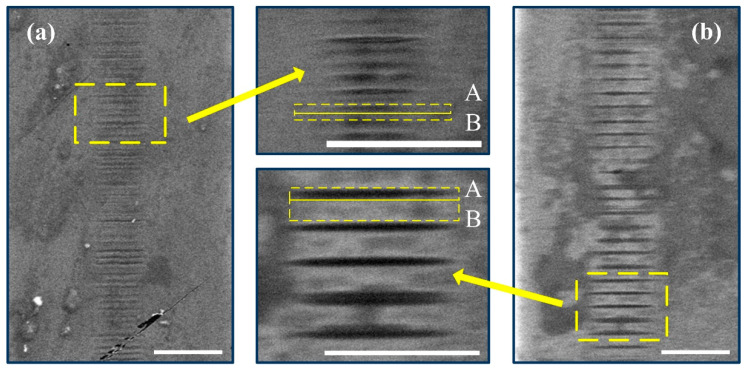
SEM images of chemically etched (15 min of etching in a 2% aqueous solution of HF acid) index modulation patterns in waveguide tracks fabricated with scanning speeds of (**a**) 50 μm/s and (**b**) 100 μm/s. Images in the middle are detailed views. Scale bar in etched image is 10 μm. The laser was run from the top.

In order to clearly observe the spatial period of the microstructures, SEM images of waveguide tracks were captured after acidic corrosion treatment, as shown in Figure 7. The etched grating planes are the isolated narrow, sheet-like cavities with a thickness of a few hundred nanometers and a lateral width equal to the waveguide diameter. The etch rate of the modulated regions with high and low refractive index change is different [23]. It is known that the etch rate of femtosecond laser modified silica structure is approximately linearly dependent on the induced index of refraction change, at least up to index changes of = 0.01 [23]. Erosion degree by acid etching increases when more structural defects appear in the focal volume. The most common defects produced in type I waveguide are the Si dangling bond with a trapped electron and nonbridging oxygens with unpaired electrons originating from laser-induced bond breaking [24]. Therefore, in Figure 7, the etched groove in region A stands for higher index change with many structural defects, while material present in region B seems to be little affected by the laser exposure. The area ratio of regions A and B is one major difference among modulated patterns formed with different scanning speeds. Specifically, region B becomes larger with the increase in the scanning speed; by contrast, the increase in the area of region A is relatively slow. In consequence, the index change in the waveguide track is periodically present. The two different etch rates in Figure 7 are mainly caused by the number of structural defects. There are more structural defects in the laser spot center, as the excitation is at maximum, while the polishing depth is hard to control because of invisible waveguide tracks by the ordinary microscope.

### 3.3. Discussion on the Mechanism for the Formation of Periodic Patterns

**Figure 8:**
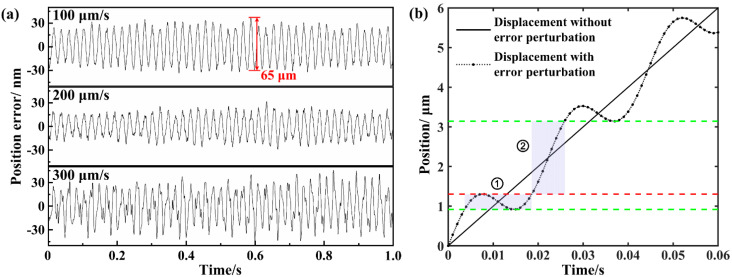
(**a**) Position errors of the motion stage with different working speeds; (**b**) displacement curve at the speed of 100 μm/s varied with given motion perturbation. The displacement perturbation in the dash-dot line assumes a sine distribution referring to the envelope of real error with an exaggerated amplitude of 650 nm higher than 19 times the measurement value and an angular frequency of 90 Hz (90 is equal to 2 × 45, in which 45 Hz is the frequency of position error in unit time shown in (**a**)).

We indicated the formation of periodic patterns along the waveguide track using a reshaped femtosecond laser beam. Even though in many cases femtosecond-laser-induced periodic patterns depend on the polarization of laser radiation and scale with the wavelength [25], the orientation and size of the modulated patterns in our experiment are only related to beam shaping of the focal laser spot and scanning speed, i.e., the imprinting feedthrough. Therefore, the formation mechanism is not due to the electromagnetic perturbation but due to the energy delivery into a dynamic system with potential movement perturbation. In addition, we can see that the laser pulse energy has little effect on the spatial period but has a significant effect on the waveguide diameter. As higher pulse energy leads to the formation of larger modification structures, isotropic thermal diffusion extends the laser-heated region far outside the focal volume, as shown in Figure 3. Since the scanning speed is directly determining the periodicity of modulated patterns, we monitored the position error and velocity error of the Aerotech stage. Figure 8a shows the relationship between the scanning speeds and the position errors of the axis, which has the linear motion during the fabrication of straight waveguides. The frequency of position error in unit time is a constant value of ~45 Hz. This proves that position error of the stage is caused by intrinsic vibrations of the platform based on the measurement of the static displacement. The time interval between neighbor peaks of periodic position error is ~22.2 ms, which is equal to the time difference for neighbor microstructures, shown in Figure 4a. Therefore, the period of the embedded patterns increases proportionally with scanning speed. It is worth noting that the maximum distance of neighbor peaks and valleys is ~65 nm at the speed of 100 μm/s and far less than the periodicity of the modulated microstructures. Figure 8b exhibited the assumed displacement of the motion stage at the speed of 100 μm/s, whose position error was exaggerated with the sinusoidal amplitude (referring to the envelope of real error) higher than 19 times the measurement value. The motion displacement during an oscillating cycle is divided into two parts, corresponding to the shadow areas in Figure 8b. In the first area, the glass matrix is irradiated 3 times by laser pulses; in the second area, the glass matrix is irradiated only once by relatively low-spatial-density laser pulses. As a consequence, the reasonable inference is that the glass is modified with the successive formation of high and low refractive index changes in the periodic patterns. Moreover, the low-index region has more area than the higher-index region, which is in accordance with Figure 7b. In fact, the spatial spacing of the continuous pulses in our experiment is 2 nm, at the speed of 100 μm/s. Even though the actual position error of the motion stage is very small, the modified region can also be divided into enrichment zone and evacuation zone for laser pulses. Therefore, it is deduced that the small perturbation in the platform’s displacement is the trigger for the generation of periodic patterns. As the influence of motion perturbation in laser pulse space distribution will decrease, or even disappear, when the scanning speed further increases, the waveguide trace is more homogeneous without periodic patterns, as shown in Figure 4a. For example, the spatial spacing of the continuous pulses in our experiment is 60 nm, at the speed of 3000 μm/s. The actual perturbation amplitude is only about 33 nm, which has little effect on the uniformity of laser pulse distribution.

The periodic experiment result may seem similar to self-organized bubble patterns fabricated as a consequence of thermal origin and cumulative energy deposition by laser pulse at a frequency of 9.4 MHz [16], where the cumulative regime is evident at a repetition rate greater than 200 kHz [26]. However, recent research has shown the relaxation dynamics occur in a large time window ranging from nanoseconds to microseconds after the irradiation of fused silica by femtosecond laser, in which molecular kinetics and structural rearrangements in low-viscosity phases play important roles [27]. In addition, the characteristic timescale here is not consistent with the rapid shock and rarefaction scenario expected at high pressures [28]. Therefore, we believe the thermal relaxation dynamics is another factor in the generation of periodic microstructures [27,29]. The small position perturbation is responsible for the perturbation of laser energy accumulation and evacuation accentuated, which influences both the dose and the focal position. The position perturbation is also considered to lead to small refocusing adjustments due to the thermos-optic effect, and cause a local temperature perturbation, which leads to temperature nonuniformity dynamics process with time. From Figure 7a, we can see that the smoothness of the error curve is decreased, and the oscillation in peaks and valleys is more evident with the increase in the working speed. The influence of the displacement error at the greater scanning speed causes more nonuniform energy distribution in the writing path and further large structural change in the long-term behavior of the dynamical system. This is the probable reason why the region with a higher refractive-index increase in the modified periodic pattern becomes slightly wider with higher scanning speed, which is in line with the phenomenon in Figure 4a and Figure 6. More investigations need to be further carried out to elucidate the complex multiphysical mechanisms for the microstructure evolution embedded in type Ι waveguide written by femtosecond laser during thermomechanical processing.

## 4. Conclusions

In summary, we described a systematic investigation on the formation of periodic index microstructures embedded in type I waveguide inside fused silica by the slit-shaped femtosecond laser. The morphology of modulated microstructures can be effectively controlled by changing laser process parameters. This was observed in SEM images following polishing and HF chemical etching. Platform perturbation during the energy delivery and dynamic variation in the accumulated dose were considered as major factors for refractive index periodic redistribution. This work can provide new insights into the interaction between ultrafast lasers’ pulse and transparent materials. However, more investigations need to be performed to elucidate the complete mechanisms underlying the periodic index modulation embedded type I waveguides.
